# Multiple mechanisms of dimethyl fumarate in amyloid β‐induced neurotoxicity in human neuronal cells

**DOI:** 10.1111/jcmm.13358

**Published:** 2017-10-09

**Authors:** Michela Campolo, Giovanna Casili, Marika Lanza, Alessia Filippone, Irene Paterniti, Salvatore Cuzzocrea, Emanuela Esposito

**Affiliations:** ^1^ Department of Chemical, Biological, Pharmaceutical and Environmental Sciences University of Messina Messina Italy; ^2^ Department of Pharmacological and Physiological Science Saint Louis University St. Louis MO USA

**Keywords:** Alzheimer's disease, dimethyl fumarate, tau hyper‐phosphorylation, Nf‐kB, oxidative stress, Nrf2

## Abstract

Alzheimer disease (AD) is characterized by a complex heterogeneity of pathological changes, and any therapeutic approach categorically requires a multi‐targeted way. It has been demonstrated that together with the hallmarks of the disease such as neurofibrillary tangles and senile plaques, oxidative and inflammatory stress covered an important role. Dimethyl fumarate (DMF) is an orally bioavailable methyl ester of fumaric acid and activator of Nrf2 with potential neuroprotective and immunomodulating activities. Therefore, the aim of the present work was to evaluate the potential beneficial effects of DMF, compared with its active metabolite monomethyl fumarate (MMF) (both at 30 μM) in an *in vitro* Alzheimer's model using SH‐SY5Y human neuroblastoma cell lines stimulated with amyloid‐beta (Aβ). Moreover, the effect of DMF, compared with MMF, was evaluate by an *ex vivo* model using organotypic hippocampal slice cultures stimulated with Aβ_1‐42_ (1 μg/ml), to better understand its action in a pathological setting. In both models, DMF pre‐treatment (30 μM) preserved cellular viability from Aβ stimulation, reducing tau hyper‐phosphorylation, much more efficiently then MMF (30 μM). Moreover, DMF was able to induce an activation of manganese superoxide dismutase (MnSOD) and heme‐oxygenase‐1 (HO‐1), decreasing the severity of oxidative stress. Our results showed important multi‐protective effects of DMF pre‐treatment from Aβ stimulation both in *in vitro* and *ex vivo* models, highlighting an Nrf2/NF‐κB‐dependent mechanism, which could provide a valuable support to the therapies for neurodegenerative diseases today.

## Introduction

Alzheimer's disease (AD) is a chronic and progressive neurodegenerative disorder, which accounts a worldwide prevalence 1, 2. The neuropathological hallmarks of the AD brain consist of diffuse neuritic extracellular β‐amyloid (Aβ) plaques, frequently surrounded by dystrophic neuritis, and hyper‐phosphorylated tau protein accumulation in intracellular neurofibrillary tangles (NFTs) at cerebral level 3; these key features are often accompanied by the presence of reactive microgliosis and the loss of neurons, grey matter and synapses. It is well studied that the neuropathological changes in AD might be the result of many altered physiological processes occurring at whole‐organism level 4. AD characteristically produces a remarkably memory loss resulting in behavioural deficits, such as disorientation in time and space and impairments in language skills. Mounting evidence suggests that these cognitive and motor deficit begin with subtle alterations of hippocampal synaptic efficacy prior to frank neuronal degeneration 5. Among the causes of this neuronal loss, certainly there is β‐amyloid and tau protein accumulation that contribute to the synaptic dysfunction 6. AD currently accounts nearly 47 million people, with a huge‐associated global cost, thereby constituting a major health and economic issue worldwide 7. At the present time, no effective disease‐modifying therapies are available in clinical practice. The approved treatments available for AD are limited to symptomatic management and consist mostly of acetylcholinesterase inhibitors (AChE) and N‐methyl‐D‐aspartate (NMDA) receptor antagonist, palliatives agents that alleviate the cognitive and functional deficits for a limited time 8. One of the alternative therapeutic approaches that are currently gaining more acceptances is based on the conception of AD pathogenesis as a pathological network that involves the simultaneous modulation of several key biological targets. Indeed, AD is a heterogeneous and progressive neurodegenerative disease, which has been linked to inflammation and oxidative stress 9. Inflammatory mechanisms clearly occur in pathologically vulnerable regions of the brain through multiple pathways, the activation of complement system and the production of cytokines and chemokines, involving phenotypic and functional responses of microglia, astrocytes and neurons 10. Research on AD has demonstrated compelling evidence on the fact that oxidative stress is closely associated with a subtle inflammatory process, highlighting that reactive oxygen and nitrogen species disrupt nerve terminals activity causing dysfunction and loss of synapses, causing hallmark features of the disease, such as neurofibrillary tangles and senile plaques 11. In the light of the complexity of the pathology and noting that multi‐target and cocktail drugs substantially reduced rates of clinical worsening 12, the multi‐target ligands, acting as master regulators of cellular defence mechanisms, may be work synergistically to exert their efficacy in a holistic way.

Between these multi‐target ligands, it has been recognized the fumaric acid esters (FAEs), a class of molecules with anti‐inflammatory and antioxidative activities in a variety of tissues and cell types, where DMF represents the most pharmacologically effective molecule among the FAEs 13. DMF is considered a prodrug because after administration, DMF is in few time cleaved into monomethyl fumarate (MMF) and fumarate *via* esterase inside cells into the small intestine DMF 14 and MMF have half‐lives of 12 min. and 36 hrs, respectively. Peak concentrations of MMF are achieved within 5–6 hrs. The parent compound does not show protein binding, although MMF is about 50% bound 15. Metabolism of MMF is through the citric acid cycle leading to excretion through respiration with no known metabolism by the cytochrome P450 system 14, 15. Currently, DMF is an oral therapeutic agent for the treatment of relapsing forms of multiple sclerosis 16. It has been shown that DMF and MMF act on Kelch‐like ECH‐associated protein 1 (Keap1), nuclear factor (erythroid‐derived 2)‐like 2 (Nrf2) activator, which works on both antioxidant and inflammatory pathways 17, promoting the attenuation of pro‐inflammatory cytokine production 18 and the modulation of microglia and astrocytes 19. It is well recognized that, in inflammatory status associated with neurodegenerative pathology, Nrf2 activity is influenced by nuclear factor kappa‐light‐chain enhancer of activated B cells (NF‐κB) pathway, more readily activated in oxidative environments 20.

Different researches have been demonstrated that oxidative stress plays a pivotal role in AD pathogenesis 21, occurring at early stages of AD, increasing Aβ production and subsequently Aβ aggregation that, as a vicious circle, further induces and exacerbates oxidative damage, encouraging Aβ toxicity and neurodegeneration 22. Several reports have shown a wide variety of antioxidants functioning as effective AD modulators, promising results in *in vitro* and *in vivo* AD models 23, 24. Furthermore, a wide array of AD‐relevant physiological stressors, including Aβ peptides and reactive oxygen and nitrogen species (ROS/RNS), stimulates NF‐κB activation, playing pathogenic roles in AD process 25.

It has been shown that post‐mortem studies of brain tissue from patients with AD provided a relevant increase in NF‐κB activity in cells involved in the neurodegenerative process: p65 immunoreactivity increases in neurons and astrocytes close to amyloid plaques in brain sections from patients with AD 26. Moreover, immunohistochemical analysis has suggested that levels of NF‐κB activity are increased in cholinergic neurons in the basal forebrains of patients with AD where its dysfunction and degeneration contribute greatly to cognitive impairment in AD 27.

Therefore, ascertaining that oxidative stress and inflammatory process are driving force in AD pathology and that Nrf2 activators and anti‐NF‐κB strategies are considered as network medicines in multiple neurodegenerative diseases and surely efficacious AD treatment strategy 28, in this study we evaluated the role of DMF and MMF, through NF‐κB‐Nrf2 signalling pathways, in an *in vitro* model of AD in SH‐SY5Y cells. These well‐differentiated cells express features specific to mature neurons, such as synaptic structures and functional axonal vesicle transport, making this new concept for *in vitro* differentiation valuable for many neuro‐scientific research areas, including AD. To evaluate the effect of DMF in a pathological setting, we performed an *ex vivo* model using organotypic hippocampal slices. Moreover, to corroborate an Nrf‐2‐dependent mechanism, a small interfering RNA system was used.

## Materials and methods

### SH‐SY5Y cell cultures

SH‐SY5Y cells are a cloned subline of SK‐N‐SH cells originally established from a bone marrow biopsy of a neuroblastoma patient with sympathetic adrenergic ganglial origin 29. SH‐SY5Y neuroblastoma cells can be differentiated into neuron‐like cells displaying morphological and biochemical features of mature neurons. Furthermore, these cells display axonal expression of mature tau protein isoforms. In the light of this, we found the best overall neuronal differentiation was achieved using retinoic acid (RA) pre‐treated SH‐SY5Y cells as previously described 30. Human neuroblastoma SH‐SY5Y cells were obtained from American Type Culture Collection (ATCC CLR‐2266) and were grown to monolayer in a culture medium containing Dulbecco's Minimal Essential Medium (DMEM) and Ham's F12; modified with 2 mM L‐glutamine, 1.0 mM sodium piruvate; and supplemented with foetal bovine serum (FBS) to 10%, streptomycin 50 mg/ml. SH‐SY5Y cells were maintained at 37°C and 5% CO_2_. For cell viability, 3 × 10^4^ cells were plated in 96‐well plates (Corning Cell Culture) in a volume of 150 μl. Progressive dilutions of DMF (1‐10‐30‐50‐100 μM) 31 were used to establish the effective concentration of DMF on cell viability, using 3‐(4,5‐dimethylthiazol‐2‐yl)‐2,5‐diphenyltetrazolium bromide (MTT) colorimetric assay. For another set of experiments, 8 × 10^5^ cells were plated and differentiated with RA (100 nM) for 24 hrs. Differentiated SH‐SY5Y cells were pre‐treated for 2 hrs with DMF 1, 10, 30 μM, respectively; then, SH‐SY5Y cells were stimulated with Aβ_1‐42_ 1 μM for 24, as previously described 32, for Western blot analysis and biochemical assay.

SH‐SY5Y cell cultures were divided into three experimental groups:

1. CTR, cells cultured with normal culture medium;
2. Aβ_1‐42_, cells stimulated with Aβ_1‐42_ (1 μM);
3. Aβ_1‐42_+MMF 30 μM: cultures stimulated as described and DMF placed in culture medium 2 hrs before Aβ_1‐42_ stimulation;
4. Aβ_1‐42_+DMF 30 μM: cultures stimulated as described and DMF placed in culture medium 2 hrs before Aβ_1‐42_ stimulation.

### Organotypic hippocampal slice cultures preparation and treatment

All experiments were performed in accordance with the National Institutes of Health guidelines for the care and use of laboratory animals and those of the Italian Ministry of Health (DL 116/92). Organotypic hippocampal slice cultures were prepared as described by Pellegrini‐Giampietro *et al*. 33 with some modifications. Briefly, CD1 mice 6 days postnatal (CD1, Harlan, Milan, Italy) were killed by decapitation and the brains removed. Coronal sections of 400 μm thickness containing the hippocampi were transversely cut using a McIlwain Tissue Chopper 34. Slices were placed into semiporous inserts (Millipore, Billerica, MA, USA) and cultured in Petri dishes with 2 ml of Minimal Essential Medium modified with 25% of Basal Medium Eagle, 25% heat‐inactivated horse serum, 10 ml HEPES (20 mM), 65%, 6.5% glucose, and 5 ml of glutamine (2 mM). Organotypic hippocampal slices were incubated at 37°C for 21 days, and the medium changed 3 times weekly. On day 21, the slices were pre‐treated with DMF and MMF at 30 μM and then stimulated with Aβ_1‐42_ (1 μg/ml) 35 and divided into the following four groups:

1. CTR: slices cultured with normal culture medium;
2. Aβ_1‐42_: slices stimulated with Aβ_1‐42_ (1 μg/ml);
3. Aβ_1‐42_+ MMF (30 μM): slices were stimulated with Aβ_1‐42_ and pre‐treated with MMF at the concentration of 30 μM;
4. Aβ_1‐42_+ DMF (30 μM): slices were stimulated with Aβ_1‐42_ and pre‐treated with DMF at the concentration of 30 μM.

### Small interfering RNA transfection

Cells were transfected with 20 nM siRNA against Nrf2 or 20 nM control siRNA (Qiagen, Hilden, Germany) for 48 hrs using Lipofectamine RNAiMAX transfection reagent (Life Technologies, Milan, Italy) following the manufacturer's instructions as previously described 36.

### Reverse Trascriptase–PCR

Total RNA (2 μg) isolated from SH‐SY5Y (4.5  ×  105 cells on a 6‐cm dish) was reverse transcribed, and synthesized cDNA was used as a template for PCR. RT‐PCR was performed on a T100 Thermal Cycler (Bio‐Rad Hercules, California, USA) with Taq polymerase (Life Technologies). cDNAs underwent 30 cycles for Nrf‐2 and GAPDH, each one performed at 94°C for 1 min., melting temperature (Tm)°C for 45 sec. and 72°C for 55 sec. (Table 1). After this treatment, 10 μl of RT‐PCR products was separated by 1.5% agarose gel electrophoresis in Tris/Borate/EDTA (TBE) 0.5 ×(Tris‐base 0.089 m, boric acid 0.089 m) containing 0.1 μg/ml of ethidium bromide. Fragments of DNA were seen under ultraviolet light. The primer sets shown in Table 1 were used to detect specific PCR products, and their values were calculated as fold change relative to control after normalization to the GAPDH gene.

**Table 1:** Primers used for detection of Nrf2 and GAPDH in human SH SY5Y cell line by reverse transcriptase‐PCR

Species	Gene	Forward primer (3′ → 5′)	Reverse primer (5′ → 3′)	bp
Humans	Nrf‐2	TACTCCCAGGTTGCCCACA	CATCTACAAACGGGAATGTCTGC	91
	GAPDH	AATGACCCCTTCATTGAC	TCCACGACGTACTCAGCGC	191

### Preparation of aggregated Aβ_1‐42_

Aβ_1‐42_ was dissolved in sterile phosphate‐buffered saline, pH 7.4 (PBS) at a concentration of 1 mM, and incubated in a Sonicator Bath at RT for 15–30 min. to induce aggregation. After aggregation, the solution was stored at −20°C until use. Immediately before treating the cells, stock solution was diluted to 1 μM final concentration in culture medium.

### Cell viability assay (MTT Assay)

The cellular viability of SH‐SY5H cells and organotypic hippocampal slice cultures was assessed using a mitochondria‐dependent dye for live cells (tetrazolium dye; MTT) to formazan, as previously described 37. Cultures are pre‐treated with increasing concentrations of the test compound and incubated at 37°C with MTT (0.2 mg/ml) for 1 hr. The medium was removed and the cells lysed with dimethyl sulfoxide (100 μl). The extent of reduction in MTT to formazan was quantified by measurement of optical density at 550 nm with a microplate rider.

### β‐Amyloid ELISA kit

The levels of Aβ _1‐42_ were performed by ELISA kit as previously described 38. In brief, microtiter plates (Maxisorp; Nunc, Termo Fisher Scientific, Waltham, MA, USA) were sensitized with streptavidin (Roche Biochemicals, Roche Diagnostics S.p.A. Monza (MB), Italy) overnight. Primary capture antibody, biotinylated 6E10 (1 mg/ml; Senetek, Maryland Heights, MO, USA) was added for 8 hrs. Concentrated (50‐fold) cell culture supernatants were diluted to 1 mg/ml with assay buffer [50 mM Tris‐HCl (pH 7.5)] containing 140 mM NaCl, 5 mM EDTA, 0.05% Nonidet P‐40, 0.25% gelatin and 1% bovine serum albumin and incubated for 24 hrs at 4°C. The BAP‐15 antibody specific for Aβ_1–42_ was used. After colour development with tetramethylbenzidine (Roche Biochemicals), the plate was analysed on a Lab systems Multiskan RC plate reader using Genesis software (Lab systems). The test was performed in triplicate.

### Tau‐ELISA kit

Phosphorylated tau levels were determined by a solid‐phase, non‐competitive sandwich ELISA as previously described 38. The test was performed in triplicate both for SH‐SY5H cells and organotypic hippocampal slice cultures.

### Western Blot Analysis

Western blot analysis was performed as previously described 39. SH‐SY5Y cells were washed for two times with ice‐cold phosphate‐buffered saline (PBS) harvested and resuspended in Tris‐HCl 20 mM pH 7.5, NaF 10 mM, 150 μl NaCl, 1% Nonidet P‐40 and protease inhibitor cocktail (Roche). After 40 min., cell lysates were centrifuged at 16000 g for 15 min. at 4°C. Protein concentration was estimated by the Bio‐Rad protein assay using bovine serum albumin as standard. Samples were heated at 95°C for 5 min., and the same amounts of protein separated on 12% SDS‐PAGE gel and blotted to a PVDF membrane (Immobilon‐P). The membrane was incubated overnight at 4°C with: anti‐Manganese SOD (MnSOD) (1:500, Millipore); anti‐Heme Oxigenase (HO) (1:500, Santa‐Cruz Biotechnology, Dallas, Texas, USA); anti‐p‐tau (1:500, Abcam); anti‐Nrf2 (1:500, Abcam); anti‐Nf‐kB (1:500, Santa‐Cruz Biotechnology); anti‐IkBα (1:500, Santa‐Cruz Biotechnology). The signals were detected with a chemiluminescence detection system reagent according to the manufacturer's instructions (Super Signal West Pico Chemiluminescent Substrate, Pierce Thermo Scientific, Rockford, IL. USA). Relative expression of bands for HO‐1 (approximately 32 kD), IκBα (approximately 37 kD), Keap‐1 (approximately 65 kD), MnSOD (approximately 24 kD), NF‐κB (approximately 65 kD), Nrf‐2 (approximately 110 kD) and p‐Tau (approximately 70 kD) was imported to analysis software (Image Quant TL, v2003); moreover, to ascertain that blots were loaded with equal amounts of protein lysate, they were also incubated with the antibody β‐actin (1:500; Santa Cruz Biotechnology), lamin A/C (1:500; Santa Cruz Biotechnology) and tau (1:500; Abcam). The relative expression of the protein bands was calculated by densitometry with Bio‐Rad ChemiDoc™ XRS + software. Molecular weight standards (10–250 kD) were used to define molecular weight positions, and as reference concentrations for each protein, as previously described 39.

### Measurement of reduced and oxidized glutathione (GSH/GSSG)

The ratio of reduced glutathione (GSH) and oxidized glutathione (GSSG) was measured in cell culture supernatant by the GSH assay kit (Cayman) using enzymatic recycling, as previously described 40. Absorbance was measured at 405 nm. The GSH/GSSG concentration of each sample was calculated as μmol/g protein.

### Determination of malondialdehyde (MDA) levels

SH‐SY5Y cells (1 × 10^5^ cells/well) were seeded in poly‐L‐lysine‐coated six‐well plates. The cells were harvested to detect the levels of malondialdehyde (MDA) using the MDA assay kit as previously described 41.

### Determination of intracellular ROS

Intracellular ROS was detected using the total ROS detection kit as previously showed 41. After various treatments, SH‐SY5Y cells and organotypic hippocampal slice cultures were trypsinized and then washed twice with 1× washing buffer. Subsequently, the cells were incubated with 5‐(and‐6)‐carboxy‐2′,7′‐dichlorodihydrofluorescein diacetate (carboxy‐H2DCFDA; 10 μM final concentration) at 37°C in the dark for 30 min. The fluorescence microplate reader detected the light emission. The level of intracellular ROS was expressed as the percentage of the control (nmol/mL).

### Materials

Unless otherwise stated, all compounds were acquired from Sigma‐Aldrich (Saint Louis, Missouri, USA). All other chemicals were of the highest commercial grade available. All stock solutions were prepared in non‐pyrogenic saline (0.9%NaCl, Baxter, Milan, Italy).

### Statistical evaluation

All values, in the figures, were evaluated as mean ± S.E.M. Results revealed in the figures are representative of at least three experiments made on different *in vivo* experimental days. The results were examined by one‐way analysis of variance followed by a Bonferroni post hoc test for multiple comparisons. A *P* value of <0.05 was considered significant.

## Results

### Protective effects of DMF pre‐treatment on viability of human SHSY5Y neuronal cells stimulated with Aβ

**Figure 1:**
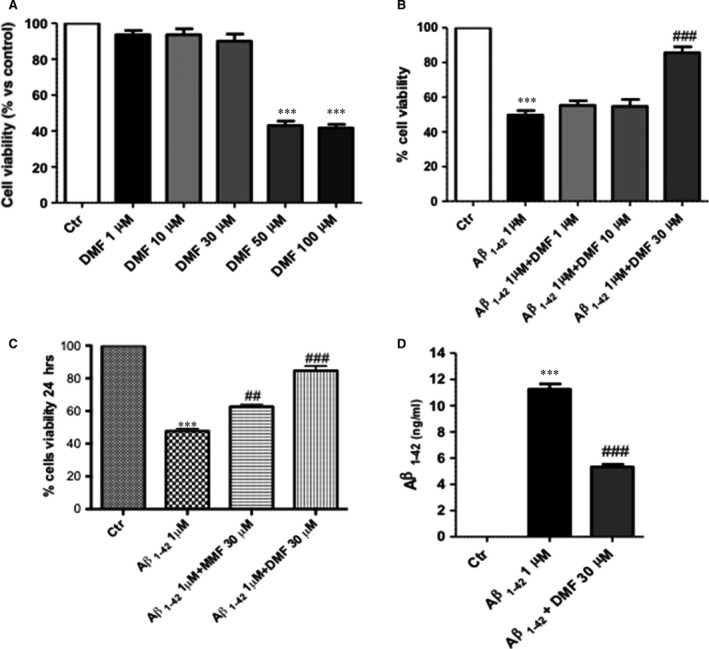
Dimethyl fumarate (DMF) pre‐treatment protected human SHSY5Y neuronal cells by Aβ_1‐42_‐induced cell death. Cell death was assessed 24 hrs after incubation with the indicated concentrations of DMF (1‐10‐30‐50‐100 μM), underlying that only DMF 1‐10‐30 μM lacked cytotoxicity (**A**). Stimulation of cells with Aβ_1‐42_ 1 μM significantly reduced viability compared to the control group and only pre‐treatment with DMF 30 μM significantly limited this cell death (**B**). MTT assay compared DMF and MMF both at the dose of 30 uM. MMF reduced partially cell death, while DMF confirmed having the greatest effect to preserve cell viability (**C**). Averages for MTT: 84,6% DMF *versus* 47,6% Aβ_1‐42_; 62,6% MMF *versus* 47,6% Aβ_1‐42_. Also, DMF 30 μM pre‐treatment determined a considerable reduction in the Aβ_1‐42_ amount, in cells 24 hrs after Aβ stimulation (**D**). Data are representative of at least three independent experiments. (**A**)***P < 0.001 *versus* Ctr; (**B**) ***P < 0.001 *versus* Ctr and ^###^ *P* < 0.001 *versus* Aβ_1‐42_; (**C**) ***P < 0.001 *versus* Ctr, ^##^ *P* < 0.01 *versus* Aβ_1‐42_ and ^###^ *P* < 0,001 *versus* Aβ_1‐42_; (**D**) ***P 0.001 *versus* Ctr and ^###^ *P* < 0.001 *versus* Aβ_1‐42._

To evaluate the effect of DMF treatment on cell viability, SH‐SY5Y cells were incubated with increasing concentrations of DMF (1‐10‐30‐50‐100 μM). Cell viability assessed after 24 hrs, DMF at the concentration of 50 and 100 μM showed mortality equal to 60%, while the concentrations of 1, 10 and 30 μM showed a viability almost comparable to control group (Fig. 1A). The fibrils of Aβ_1–42_ is commonly found in the senile plaques in AD brains to cause neuronal death 42, so we examined the effects of DMF pre‐treatment on SH‐SY5Y cells stimulated with fibril Aβ_1–42_ for 24 hrs. Incubation of SH‐SY5Y cells with Aβ_1‐42_ 1 μM after 24 hrs significantly reduced cells viability and translated in a decreasing of MTT metabolism (Fig. 1B). Pre‐treatment with DMF 30 μM, 2 hrs before Aβ_1‐42_, significantly reduced cell death compared to the Aβ_1‐42_ group, while DMF 1 and 10 μM were not able to reduce Aβ_1‐42_‐induced cell death (Fig. 1B), thus demonstrating DMF 30 μM to be the most effective concentration. Moreover, compared to DMF 30 μM (85%), the pre‐treatment with MMF 30 μM was able to preserve partially cell viability (67%) (Fig. 1C).

Furthermore, Aβ‐ELISA kit was performed to quantify the reduction in Aβ_1‐42_ in DMF‐pre‐treated neurons. Pre‐incubation of SHSY5Y cells with DMF 30 μM resulted in a considerably reduced amount of Aβ_1‐42_ in cells at 24 hrs after Aβ damage (Fig. 1D) compared to Aβ_1‐42_‐stimulated group (Fig. 1D).

### Effects of DMF on Aβ‐induced tau phosphorylation in human SHSY5Y neuronal cells

**Figure 2:**
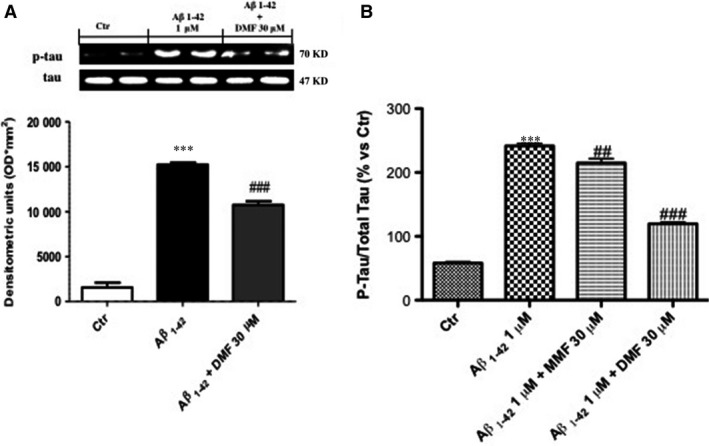
Dimethyl fumarate (DMF) pre‐treatment preserved Aβ‐induced tau phosphorylation in human SHSY5Y neuronal cells. As seen by Western blot analysis of p‐tau, incubation with Aβ_1‐42_ 1 μM significantly increased p‐tau after 24 hrs of stimulation (**A**), while pre‐treatment with DMF 30 μM for 2 hrs effectively prevented the increase in phosphorylation (**A**). Comparably, as measured by tau‐ELISA kit, pre‐treatment with DMF 30 μM for 2 hrs notably reduced the quantity of tau‐phosphorylated protein, more efficiently than MMF 30 μM (**B**). Averages for p‐tau‐ELISA kit: 119.6% *versus* 241,6% Aβ_1‐42;_ 214,8% MMF *versus* 241,6% Aβ_1‐42_. Data are representative of at least three independent experiments. (**A**) ***P 0.001 *versus* Ctr and ^###^ *P* < 0.001 *versus* Aβ_1‐42_; (**B**) ***P 0.001 *versus* Ctr, ^##^ *P* < 0.01 *versus* Aβ_1‐42_ and ^###^ *P* < 0.001 *versus* Aβ_1‐42_.

It is known that increment in Aβ‐induced tau phosphorylation is a key aspect of AD 43; to evaluate whether the neuroprotective effect of DMF is also related to tau phosphorylation, we examined the expression of phosphor‐tau (p‐tau) in SHSY5Y cells. As shown by Western blot analysis of p‐tau, incubation with Aβ_1‐42_ 1 μM significantly increased tau phosphorylation 24 hrs after stimulation compared to control group (Fig. 2A). Pre‐treatment with DMF 30 μM for 2 hrs effectively reduced p‐tau expression (Fig. 2A). Also, to confirm the reduction in tau phosphorylation mediated by DMF, tau‐ELISA kit was performed. Aβ stimulation significantly increased phosphorylation levels of tau protein 24 hrs after incubation (Fig. 2B), while pre‐treatment with DMF 30 μM notably reduced the quantity of tau‐phosphorylated protein, more efficiently than MMF 30 μM (Fig. 2B).

### Modulatory effects of DMF pre‐treatment on Nrf2‐mediated antioxidant response

**Figure 3:**
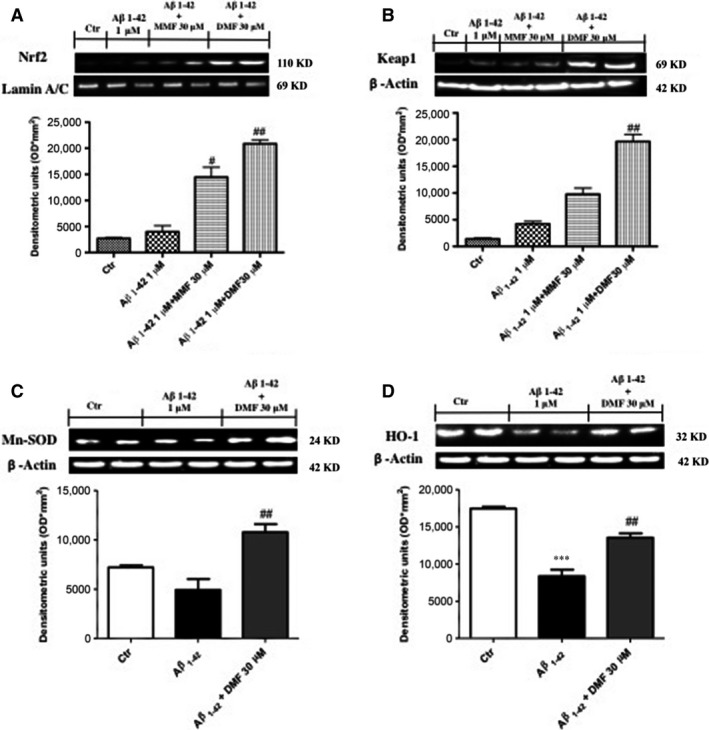
Dimethyl fumarate (DMF) pre‐treatment on Nrf2‐mediated antioxidant response. Nrf2 expression showed a tendency to decrease following Aβ_1‐42_ 1 μM stimulation (**A**), while DMF 30 μM pre‐treatment up‐regulated Nrf2 levels as compared to control group, much more than pre‐treatment with MMF 30 μM pre‐treatment (**A**). Western blot analysis of Keap 1 showed low expression in the control group, and after Aβ_1‐42_ stimulation, the levels of Keap1 were partially increased after MMF pre‐treatment, while the pre‐treatment with DMF significantly increase Keap 1 expression (**B**). Comparable decreases in levels of both MnSOD and HO‐1 expression (**C** and **D**, respectively) after 24 hrs of Aβ_1‐42_ 1 μM incubation. Pre‐treatment with DMF 30 μM up‐regulated MnSOD expression (**C**), with a tendency to report HO‐1 expression at control values (**D**). Data are representative of at least three independent experiments. (**A**) ^##^ *P* < 0.01 *versus* Aβ_1‐42_; (**B**) ^##^ *P* < 0.01 *versus* Aβ_1‐42_; (**C**) ^##^ *P* < 0.01 *versus* Aβ_1‐42_; (**D**) ***P 0.001 *versus* Ctr and ^##^ *P* < 0.01 *versus* Aβ_1‐42_.

A major sign of ageing is oxidative stress, and a significant amount of evidence has shown that oxidative stress is an important pathogenic factor in AD 23 and DMF, as Nrf2 activator, acts as master regulator to activate a cellular defence process to protect neurons from ROS‐induced damage 44. Thus, we evaluated the effect of DMF on the Nrf2 pathway by Western blot analysis. Nrf2 expression showed a tendency to decrease following Aβ_1‐42_ 1 μM stimulation (Fig. 3A). MMF increased partially Nrf2 expression, while pre‐treatment with DMF 30 μM significantly up‐regulated Nrf2 levels almost analogous to control group (Fig. 3A).

Keap 1 covers an important role in the Nrf‐2 signalling pathway that regulates DMF activity 45. Western blot analysis showed low levels of Keap1 in the control group as well as following Aβ_1‐42_ stimulation; the levels of Keap1 were partially increased after MMF pre‐treatment, while the pre‐treatment with DMF markedly increases Keap 1 expression (Fig. 3B, seed densitometric analysis).

Moreover, there was a comparable decrease in levels of both MnSOD and HO‐1 expression (Fig. 3C and D, respectively) 24 hrs after Aβ_1‐42_ incubation. Interestingly, pre‐treatment with DMF 30 μM up‐regulated MnSOD and HO‐1 expression (Fig. 3C and D, respectively).

### The role of DMF pre‐treatment on oxidative stress‐related indicators

**Figure 4:**
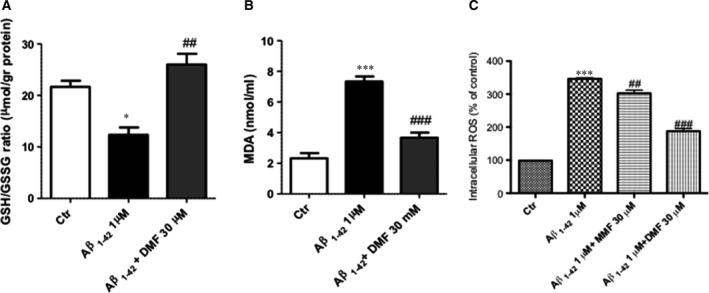
Dimethyl fumarate (DMF) pre‐treatment on oxidative stress‐related indicators. A considerable reduction in the level of GSH/GSSG ratio was observed in Aβ_1‐42_‐stimulated SHSY5Y cells after 24 hrs of incubation (**A**) compared to control group, while pre‐treatment with DMF 30 μM considerably increased GSH/GSSG ratio (**A**). Furthermore, pre‐treatment with DMF 30 μM for 2 hrs notably prevented the elevated lipid peroxidation caused by Aβ_1‐42_ stimulation (**B**) and significantly reduced intracellular ROS concentrations increment Aβ_1‐42_‐induced, more than MMF 30 μM (4). Averages for ROS assay: 187% DMF *versus* 346% Aβ_1‐42_; 302% MMF *versus* 346% Aβ_1‐42_. Data are representative of at least three independent experiments. (**A**) *P 0.05 *versus* Ctr and ^##^ *P* < 0.01 *versus* Aβ_1‐42_; (**B**) ***P 0.001 *versus* Ctr and ^###^ *P* < 0.001 *versus* Aβ_1‐42_; (**C**) *** *P* 0.001 *versus* Ctr, ^##^ *P* < 0.01 *versus* Aβ_1‐42_ and ^###^ *P* < 0.001 *versus* Aβ_1‐42_.

The measurement of glutamate‐induced oxidative stress is an indicator of cellular health, with considerable interest in Alzheimer's disease 46, 47, so to further investigate the role of DMF against Aβ_1‐42_‐induced neurotoxicity correlated with oxidative stress, the GSH/GSSG ratio was performed by ELISA kit. A considerable reduction in the level of GSH/GSSG ratio was observed in Aβ_1‐42_‐stimulated SHSY5Y cells 24 hrs after incubation (Fig. 4A) compared to control group, while pre‐treatment with DMF 30 μM considerably increased GSH‐reduced levels, close to control conditions (Fig. 4A). Furthermore, it is well known that malondialdehyde (MDA), one of the products of membrane lipid peroxidation, reflects the degree of oxidative stress‐inflicted damage *via* membrane lipid peroxidation 48; therefore, MDA assay was performed by ELISA kit. Two hours of pre‐treatment with DMF 30 μM notably prevented the elevated lipid peroxidation caused by Aβ_1‐42_ stimulation (Fig. 4B), bringing it to control levels (Fig. 4B). Moreover, the intracellular ROS assay, intended as the initial species generated by oxygen reduction (superoxide or hydrogen peroxide) as well as their secondary reactive products 49, was performed to assess the effects of DMF to modulate ROS production caused by Aβ_1‐42_ stimulation. The incubation for 24 hrs with Aβ_1‐42_ 1 μM resulted in a significant increase in intracellular ROS concentrations (Fig. 4C), which was significantly reduced by pre‐treatment with DMF 30 μM (Fig. 4C), much more than MMF 30 μM pre‐treatment.

### Modulatory effects of DMF pre‐treatment on NF‐κB‐mediated inflammatory response

**Figure 5:**
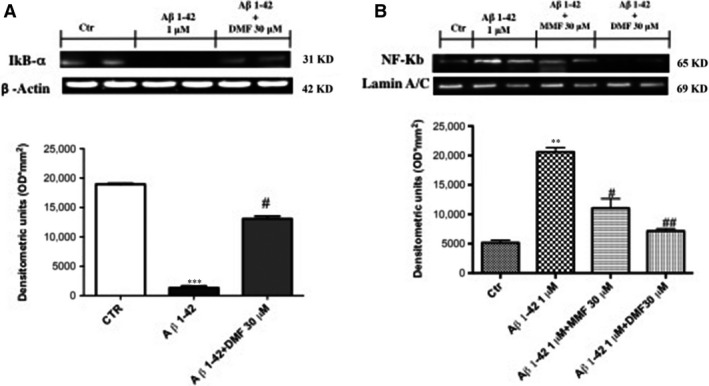
Dimethyl fumarate (DMF) pre‐treatment on Nf‐kB inflammatory pathway. IκB‐α degradation increase markedly in Aβ_1‐42_‐stimulated cells as compared to control group (**A**), while the pre‐treatment with DMF 30 μM preserved IκB‐α cytosolic activity (**A**), Aβ_1‐42_ intoxicated neurons showed an important increase in NF‐κB DNA‐binding activity as compared to control group (**B**). DMF pre‐treatment at 30 μM significantly attenuated NF‐κB nuclear translocation, more efficiently than MMF 30 μM (**B**). Densitometric analyses are shown in A1 and B1, respectively. Data are representative of at least three independent experiments. (**A**) ***P 0.001 *versus* Ctr and ^#^ *P* < 0.05 *versus* Aβ_1‐42_; (**B**) **P 0.005 *versus* Ctr, ^##^ *P* < 0.01 *versus* Aβ_1‐42_ and ^#^ *P* < 0.05 *versus* Aβ_1‐42_.

To assess the anti‐neuroinflammatory activity of DMF on Aβ_1‐42_‐induced inflammatory pathway, we evaluated the expression of NF‐κB and IκB‐α by Western blot analysis. The cells exposition with Aβ_1‐42_ considerably increased NF‐κB DNA‐binding activity as compared to control group (Fig. 5A, see densitometric analysis A1). The pre‐treatment with DMF (30 μM) significantly attenuated NF‐κB nuclear traslocation in Aβ_1‐42_‐exposed neurons, more efficiently than the treatment with MMF (30 μM) (Fig. 5A, see densitometric analysis A1). Moreover, the involvement of NF‐κB pathway is confirmed by IκB‐α degradation that was markedly increased in Aβ_1‐42_ intoxicated neurons as compared to control group (Fig. 5B, see densitometric analysis B1), while DMF (30 μM) pre‐treatment operated to maintain IκB‐α cytosolic activity (Fig. 5B, see densitometric analysis B1).

### Protective effect of DMF on viability in Aβ_1_‐42‐treated organotypic hippocampal slice cultures

**Figure 6:**
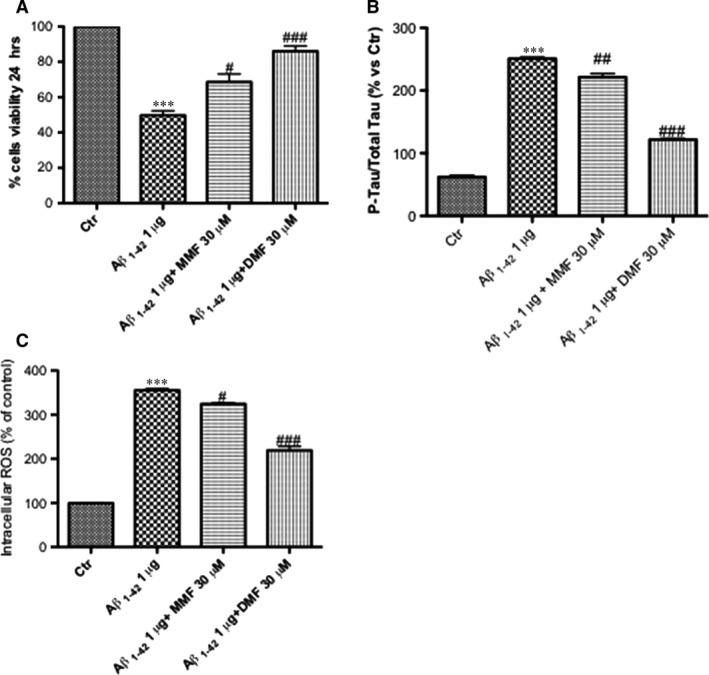
Effect of DMF on viability, p‐tau and ROS production in Aβ_1‐42_‐treated organotypic hippocampal slice cultures. Stimulation of organotypic slides with Aβ_1‐42_ significantly reduced viability compared to the control group (**A**). Pre‐treatment with DMF (30 μM), much more than MMF (30 μM), 2 hrs before Aβ_1‐42_ stimulation, significantly reduced cell death compared to the Aβ_1‐42_ group (86% *versus* 68%, respectively) (**A**). Averages for MTT: 86% DMF *versus* 49,6% Aβ_1‐42_; 68,6% MMF *versus* 49,6% Aβ_1‐42_. ELISA kit of p‐tau showed an important increase in phosphorylation after Aβ_1‐42_ stimulation 24 hrs after incubation (**B**); the pre‐treatment with DMF 30 μM notably reduced the quantity of tau‐phosphorylated protein, more efficiently than MMF 30 μM (**B**). Averages for p‐Tau‐ELISA kit: 121,6% DMF *versus* 250,2% Aβ_1‐42_; 221,4% MMF *versus* 250,2% Aβ_1‐42_. Pre‐treatment with MMF 30 μM partially reduced ROS production, while the pre‐treatment with DMF 30 μM has shown a significant reduction in ROS‐produced oxidative stress compared to Aβ_1‐42_‐stimulated group. (**C**). Averages for ROS assay: 219,3% DMF *versus* 355,6% Aβ_1‐42_; 324,3 MMF *versus* 355,6% Aβ_1‐42_. Data are representative of at least three independent experiments. (**A**) ***P < 0.001 *versus* Ctr, ^##^ *P* < 0,01 *versus* Aβ_1‐42_ and ^###^ *P* < 0.001 *versus* Aβ_1‐42_; (**B**) ***P < 0.001 *versus* Ctr, ^##^ *P* < 0.01 *versus* Aβ_1‐42_ and ^###^ *P* < 0.001 *versus* Aβ_1‐42_; (**C**) ***P < 0.001 *versus* Ctr, ^##^ *P* < 0,01 *versus* Aβ_1‐42_ and ^###^ *P* < 0.001 *versus* Aβ_1‐42_.

Incubation of slices with Aβ_1‐42_ significantly reduced viability compared to the uninjured group (Fig. 6A). Pre‐treatment with DMF (30 μM) and MMF (30 μM), 2 hrs before Aβ_1‐42_ stimulation significantly reduced cell death compared to the Aβ_1‐42_ group (Fig. 6A). However, DMF showed cell viability greater than the pre‐treatment with MMF (86% and 68%, respectively).

### Effects of DMF on Aβ‐induced tau phosphorylation in organotypic hippocampal slice cultures

To confirm the reduction in tau phosphorylation mediated by DMF, ELISA kit for p‐tau was performed on organotypic hippocampal slice cultures. Aβ stimulation significantly increased tau phosphorylation levels 24 hrs after incubation (Fig. 6B); more efficiently than MMF 30 μM, the pre‐treatment with DMF 30 μM notably reduced the quantity of tau‐phosphorylated protein (Fig. 6B).

### Pre‐treatment with DMF reduced ROS production in organotypic hippocampal slice cultures

The incubation of slices for 24 hrs with Aβ_1‐42_ 1 μg/ml brought a significant increase in intracellular ROS concentrations (Fig. 6C); pre‐treatment with MMF 30 μM partially reduced ROS production, while the pre‐treatment with DMF 30 μM has shown a significant reduction in ROS‐produced oxidative stress (Fig. 6C).

### The lack of Nrf2 abolished DMF protective effect

**Figure 7:**
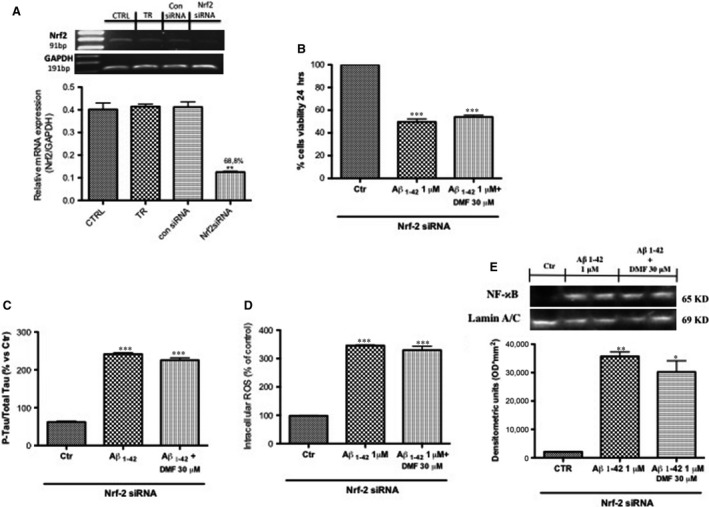
Effect of DMF in SHSY5Y following Nrf2 siRNA knockdown. Nrf2 mRNA expression in mouse SH‐SY5Y that were either transfected for 48 hrs with 20 nM control siRNA and 20 nM Nrf2‐specific siRNA, and treated with transfection reagent (TR) only or untreated cells (CTRL) was determined using real‐time PCR. Values are normalized to GAPDH and expressed as fold change to untreated control cells (**A**). The lack of Nrf2 significantly abolished DMF protective effect, increasing SH‐SY5Y susceptibility to Aβ_1‐42_ damage; in fact, the pre‐treatment with DMF was not able to preserve from Aβ_1‐42_‐induced cell death (54% and 49%, respectively) (**B**); moreover, the pre‐treatment with DMF did not protect from intracellular ROS production (**D**) and phosphorylation of tau protein (**C**). Western blot analysis has demonstrated a lost of anti‐inflammatory effect of DMF showing an Nf‐kB expression compared to Aβ_1‐42_ group (**E**). Data are representative of at least three independent experiments. (**A**) **P < 0.01 *versus* CTRL, TR and con siRNA, (**B**) ***P < 0.001 *versus* Ctr; (**C**) ***P < 0.001 *versus* Ctr; (**D**) ***P < 0.001 *versus* Ctr; (**E**) ***P < 0.001 *versus* Ctr and *P < 0,05 *versus* Ctr.

In addition, to corroborate that the anti‐inflammatory and antioxidant effects of DMF treatment are Nrf‐2 mediated, we evaluated the action of DMF treatment on SHSY5Y cells following Nrf‐2 siRNA knockdown. In human SH‐SY5Y, Nrf2‐specific siRNA successfully reduced mRNA levels by 68,8% (Fig. 7A).

The lack of Nrf2 significantly abolished DMF protective effect, increasing SH‐SY5Y susceptibility to Aβ_1‐42_ damage; in fact, the pre‐treatment with DMF was not able to preserve from Aβ_1‐42_‐induced cell death (54% and 49%, respectively) (Fig. 7B); moreover, the pre‐treatment with DMF did not protect from intracellular ROS production (Fig. 7D) and phosphorylation of tau protein (Fig. 7C). In addition, its anti‐inflammatory effect was abolished by Nrf‐2 lacking as showed by Nf‐kB expression. These data strengthen the thesis that Nrf2 inhibition antagonized the protective effect of DMF (Fig. 7E, see densitometric analysis).

## Discussion

Amyloid β‐peptide [Aβ (1–42)] is pivotal to the pathogenesis of Alzheimer's disease (AD); its involvement in neurotoxicity is referred to the aptitude to induce neuronal apoptosis by generating oxidative stress 50, 51. In fact, oxidative stress that appears within the bilayer, speculated in the Aβ‐induced oxidative stress hypothesis in which Aβ_1–42_ inserts as oligomers into the bilayer and operates as a source of ROS, has been shown to initiate also lipid peroxidation 52, 53. In tissue post‐mortems from patients with AD including the temporal cortex and hippocampus, HO‐1 expression is significantly higher as compared to non‐demented patients 54, 55. Likewise, the expression of NAD(P)H dehydrogenase [quinone] 1 (NQO1) is improved in neurons and astrocytes in patients with AD 56, 57. Furthermore, there are clear evidence that individuals with AD have shown an effective relationship with the activation of inflammatory pathways such as Nf‐kB, realizing that some components of this complex cellular and molecular machinery are very probably promoter of pathological processes leading to AD 58.

Therefore, preventing the Aβ oxidative and inflammatory toxicity may be the milestone to halting the progression of AD.

DMF is a natural powerful antioxidant recently approved from FDA as a treatment of multiple sclerosis 59.

Usually, DMF after administration is quickly metabolized to MMF by esterases in the intestine although the degree and the compartments that are directly exposed to significant levels of DMF or MMF are yet to be defined; for this reason, many preclinical studies treat DMF and MMF as interchangeable molecules and not as two structurally related but distinctive compound 60.

Recent experimental studies have shown that DMF exerts beneficial effects in preclinical models of neuroinflammation and neurodegeneration 61: *in vitro,* it has been shown to protect SH‐SY5Y cells against 6‐OHDA‐induced neurotoxicity and the brain from oxidative stress *via* Nrf‐2‐dependent mechanisms 62. Moreover, in an *in vivo* model of Parkinson, DMF significantly reduced neuronal degeneration of dopaminergic tract and behavioural impairments 31.

Here, we investigated the protective effects of DMF on Aβ‐induced oxidative toxicity compared with its active metabolite form MMF. The widespread neuronal dysfunction in the AD brain is caused by soluble Aβ aggregates, such as protofibrils, rather than the insoluble fibrils 63, 64. The approaches by which Aβ42 protofibrils provoke neuronal toxicity are multiple. It has been mainly suggested that Aβ oligomers induced neuronal cell death, inhibiting long‐term potentiation and impair synaptic function and plasticity 65, 66, 67. In the present study, we showed that pre‐incubation with DMF at the dose of 30 μM on mature neurons resulted in a significant reduction in Aβ_1‐42_ neurotoxicity by preserving cell viability and by reduction in Aβ42 protofibrils.

Aβ interrelates with the signalling pathways that modulate the phosphorylation of the microtubule‐associated protein tau. Hyper‐phosphorylation of tau interrupts its normal function in regulating axonal transport and brings to the accumulation of neurofibrillary tangles and toxic species of soluble tau 68. Therefore, these two proteins and their associated signalling pathways symbolize important therapeutic targets for AD. Our data clearly demonstrated that DMF 30 μM influenced tau phosphorylation showing an important reduction following Aβ exposition.

Newly, it was identified that insufficient Nrf2 activation in humans is linked to chronic diseases such as PD 31, 69, 70, AD 71, 72 and amyotrophic lateral sclerosis 73. Our findings provide elucidation of the molecular mechanisms involved in the neuroprotective action of DMF, evidencing the importance of the cell survival signalling pathways regulated by Nrf2 in term of oxidative stress. In fact, oxidative stimuli modify the cysteine residues of Keap‐1, thus enabling translocation of Nrf2 into the nucleus where it binds to the antioxidant responsive element (ARE) located in the promoter region of antioxidant genes, including HO‐1 and MnSOD 74. Our study showed that DMF 30 μM up‐regulates MnSOD and HO‐1 expression, *via* Nrf‐2 pathway, conferring resistance against Aβ exposition. Our findings provide new comprehensions on the biochemical properties of DMF as Nrf2/HO‐1/MnSOD activators, which are significant for the control of oxidative stress in neurons.

In response to oxidative or electrophilic stress, Nrf2 coordinately modulates not only the expression of antioxidants enzyme, but also genes associated with glutathione (GSH) pathway (glutathione peroxidase, glutathione reductase, Gclc and Gclm), thioredoxin pathway (thioredoxin reductase, peroxiredoxin) and NADPH‐regenerating enzymes 75. Moreover, it is was found that GSH content was significantly decreased in the red blood cells from male patients with AD, associated with an increase in ROS production 76. Accordingly, our results indicated clearly the reduction in GSH pathway in terms of ratio of reduced GSH and oxidized GSH (GSSG) after Aβ exposition. DMF 30 μM was able to increase significantly intracellular GSH levels through the activation of Nrf2. In addition, our data demonstrated that DMF 30 μM, *in vitro*, had a protective effect against Aβ cytotoxicity mediated by intracellular ROS.

The theory that Aβ induces lipid peroxidation, resulting in neurotoxic free radicals and reactive aldehydes, is a crucial element of the Aβ‐associated free radical model for neurodegeneration in AD 77. Therefore, the extensive lipid peroxidation in AD brain suggests that exogenous antioxidants that can inhibit free radical‐induced lipid peroxidation may be a promising therapeutic strategy in AD. In fact, pre‐treatment with DMF 30 μM reduced significantly the lipid peroxidation induced by Aβ intoxication. The relationship between Nrf‐2 and NF‐κB is well linked by the identification of NF‐κB binding sites in the promoter region of the Nrf‐2 gene suggesting crosstalk between these two regulators of inflammatory processes 78. Accordingly, the pre‐treatment with DMF 30 μM inhibited the nuclear translocation of NF‐κB and the degradation of IκB‐α in the cytosol, representing a functional system to the regulation of inflammation in response to oxidative stress.

To gain a better understanding of the neuroprotective effects of DMF in a pathological setting, we used an *ex vivo* model stimulating the hippocampal slice cultures with Aβ _1‐42_. Using this model, it is offered the advantage of preserving neuronal and glial cells within their physiological connections and long‐term survival 79. We observed that the treatment with DMF significantly reduced Aβ _1‐42_‐induced cell death. Moreover, we confirmed that Aβ _1‐42_ stimulation increased phosphorylation of tau and intracellular ROS production, while the treatment with DMF was able to reduce both parameters.

Moreover, to validate the anti‐inflammatory and antioxidant effects of DMF as an NF‐κB/Nrf‐2‐dependent molecule, we evaluated DMF capacity to protect against cell death, tau phosphorylation and ROS production after Aβ _1‐42_ stimulation in SH‐SY5Y cells lacking of Nrf‐2 gene. The obtained results highlighted that DMF treatment loses its effectiveness in the absence of Nrf‐2.

Despite these promising results, showing for the first time the protective effect of DMF on Aβ‐induced damage, further *in vivo* studies are needed to confirm these data.

It was widely ascertained that the form of 42‐amino acid of this peptide, Aβ (1‐42), to the pathogenesis of AD has been coupled to the extensive oxidative stress and inflammatory activity in the brain 52, 58; together, our results indicate that DMF operated its protective role by: 1. reduction in Tau hyper‐phosphorylation; 2. activation of MnSOD and HO‐1; 3. suppression of the Nf‐kB pro‐inflammatory effect; and 4. modulating the Nrf2 pathway. Our study clearly confirms the involvement of Nrf2 and Nf‐kB system in protecting against Aβ‐induced cytotoxicity; therefore, DMF could represent a potential therapeutic treatment for limiting the inflammatory and oxidative impact on brain neurons due to amyloid peptides exposure.

## Conflicts of interest

The authors declare no conflict of interest.
